# ERβ1 inhibits metastasis of androgen receptor-positive triple-negative breast cancer by suppressing ZEB1

**DOI:** 10.1186/s13046-017-0545-x

**Published:** 2017-06-05

**Authors:** Wei Song, Lin Tang, Yumei Xu, Qian Sun, Fang Yang, Xiaoxiang Guan

**Affiliations:** 1 Department of Medical Oncology, Jinling Hospital, Southern Medical University, Guangzhou, 510515 China; 2 Department of Medical Oncology, Jinling Hospital, Medical School of Nanjing University, Nanjing, 210002 China; 3 Department of Medical Oncology, Jinling Hospital, Nanjing Medical University, Nanjing, 210029 China

**Keywords:** ERβ1, AR, ZEB1, Triple-negative breast cancer

## Abstract

**Background:**

Increasing evidence has indicated an important role for estrogen receptor beta 1 (ERβ1) in breast cancer. However, the role of ERβ1 in the metastasis of androgen receptor (AR)-positive triple-negative breast cancer (TNBC) and the underlying mechanisms are still unknown.

**Methods:**

Stable ERβ1-expressing TNBC cell lines were generated for this study. We detected the abilities of cell migration and invasion by wound-healing and transwell assays and the expression of E-cadherin and N-cadherin by quantitative RT-PCR (qRT-PCR) and western blotting assays in TNBC cell lines. Chromatin immunoprecipitation (ChIP) analysis was performed to assess the effect of AR on ERβ1 promoter. Tumor metastasis was evaluated in vivo using a lung metastasis mouse model. Lastly, immunohistochemical expression of ERβ1 in TNBC tissues was analyzed and correlated with clinicopathological features.

**Results:**

ERβ1 suppressed the invasion and migration abilities of AR-positive TNBC cells and induced the downregulation of ZEB1. ZEB1 overexpression abrogated the increase in E-cadherin expression and the decrease in N-cadherin expression modulated by ERβ1. A lung metastasis mouse model showed that the incidence of metastasis was lower in ERβ1-expressing TNBC cells. Further, AR activation increased the anti-metastatic effect of ERβ1 in AR-positive TNBC cells, which accelerated ERβ1 transcription by functioning as a transcription factor that bound to the promoter of ERβ1. No significant change was observed in AR expression induced by ERβ1. Immunohistochemistry (IHC) analysis of TNBC clinical samples showed that ERβ1 and AR were positive in 31.7% and 23.2% of samples, respectively. ERβ1 expression was negatively correlated with ZEB1 expression and lymph node metastasis, and positively correlated with the expression of AR and E-cadherin.

**Conclusion:**

Our findings suggested a potential role of ERβ1 in metastasis of AR-positive TNBC and provided novel insights into the mechanism of action of ERβ1 and the possible relationship between ERβ1 and AR.

## Background

TNBC is a special subgroup of breast cancer characterized by lack of estrogen receptor alpha (ERα), progesterone receptor (PR) and human epidermal growth factor receptor 2 (HER2), and it accounts for approximately 15 to 20% of breast cancer patients [1]. TNBC is more aggressive than other breast cancer subtypes, and it is more likely to metastasize at an early stage [2, 3]. TNBC remains the hardest breast cancer subtype to treat because it is a highly heterogeneous disease and lacks effective targets for therapy [2]. Thus, it is important to classify effective TNBC subtypes and identify novel therapeutic targets. Approximately 12–36% TNBC patients are AR-positive [4–6]. As a newly emerging biomarker, the role of the AR pathway has been recently investigated in TNBC. Conflicting results have been reported in preclinical studies, and their impact on clinical outcome is still debated. McGhan LJ et al. declared that AR-positive TNBC has a higher propensity for lymph node metastases [7]. However, it has also been shown that there is not any significant correlation between the sites of distal metastasis and AR status in recurrent specimens [8]. Lisa MS et al. indicated that decreased AR expression is associated with distant metastases in patients with AR-expressing TNBC [9]. AR positivity has also been reported to be associated with lower risk of disease recurrence in TNBC [10].

The downregulation of ERβ1, the fully functional ERβ isoform (also known as wild-type ERβ), promotes epithelial-mesenchymal transition (EMT) in prostate cancer cells [11]. ERβ1 has also been investigated in breast cancer with contradictory results. Some studies have shown that ERβ1 inhibits the growth and decreases the invasiveness of breast cancer cells, and it predicts a favorable survival for ERα-negative breast cancer [12–14]. Whereas estrogen receptor beta 2 (ERβ2), one of the splice variants of ERβ, has been reported to be associated with poor prognosis in ERα-negative breast cancer [15]. Other studies have indicated that ERβ1 has no prognostic significance in breast cancer [16, 17]. Among TNBC patients, approximately 30% show overexpression of ERβ1 [12, 18]. Patients with TNBC harboring ERβ1-positive tumors treated with adjuvant tamoxifen have significantly better survival [19]. However, little is known about the functions and underlying mechanisms of ERβ1 in metastasis in AR-positive TNBC. In this study, stable ERβ1-expressing cells were generated using two AR-positive TNBC cell lines. ERβ1 suppressed the invasion and migration abilities of AR-positive TNBC cells by inducing the downregulation of ZEB1. This study also investigated the potential regulatory relationship between ERβ1 and AR as well as the association of ERβ1 with AR and ZEB1 in TNBC clinical samples.

## Methods

### Clinical specimens

Eighty two TNBC tissue samples were collected from patients who underwent tumor resection in The First Affiliated Hospital of Wenzhou Medical University from April 2005 to March 2014. The use of clinical tissues for this study was approved by the Jinling Hospital’s Ethics Committees and conducted in accordance with the Helsinki Declaration. All patients gave their informed consent prior to inclusion in the study.

### Cell lines, Animals and Reagents

Human TNBC cell lines, MDA-MB-231 cells and Hs578T cells were obtained from the American Type Culture Collection (ATCC, Manassas, VA, USA) and cultured in RPMI-1640 or Dulbecco’s Modified Eagle Medium supplemented with 10% fetal bovine serum (FBS) at 37 °C in 5% CO_2_. For the ligand experiments, cells were maintained in phenol red-free media containing 5% dextran-coated charcoal (DCC)-treated FBS. BALB/c athymic nude mice (female, 6 weeks old) were purchased from the Department of Comparative Medicine, Jinling Hospital (Nanjing, China) and maintained in a pathogen-free facility. 5α-Dihydrotestosterone (DHT) was purchased from Sigma-Aldrich (St. Louis, USA).

### RNA extraction and qRT-PCR

Total RNA was extracted from cultured cells using TRIzol (Invitrogen, USA). For qRT-PCR analysis, cDNA was synthesized using the PrimeScript^TM^ RT Master Mix (Perfect Real Time) Kit (RR036A, Takara, China), and PCR was performed using the Power SYBR Green PCR Master Mix (Life Technology, USA). The primer sequences were as follows: ERβ1 forward 5’- CGATGCTTTGGTTTGGGTGAT-3’ and reverse 5’-GCCCTCTTTGCTTTTACTGTC-3’; AR forward 5’-CCTGGCTTCCGCAACTTACAC-3’ and reverse 5’-GGACTTGTGCATGCGGTACTCA-3’; E-cadherin forward 5’-TGAAGGTGACAGAGCCTCTGGAT-3’ and reverse 5’-TGGGTGAATTCGGGCTTGTT-3’; N-cadherin forward 5’-CACTGCTCAGGACCCAGAT-3’ and reverse 5’-TAAGCCGAGTGATGGTCC-3’; ZEB1 forward 5’-GCCAATAAGCAAACGATTCTG-3’ and reverse 5’-TTTGGCTGGATCACTTTCAAG-3’; Snail forward 5’-CACTATGCCGCGCTCTTTC-3’ and reverse 5’-GGTCGTAGGGCTGCTGGAA-3’; Twist forward 5’-AGTCCGCAGTCTTACGAGGA-3’ and reverse 5’-GCCAGCTTGAGGGTCTGAAT-3’; and GAPDH forward 5’-AAATCAAGTGGGGCGATGCTG-3’ and reverse 5’-GCAGAGATGATGACCCTTTTG-3’. The 2^−ΔΔCt^ method was used to determine the relative mRNA expression.

### Plasmids and Transfection

The human full-length cDNA of ERβ1 and ZEB1 were cloned into the pFLAG-CMV expression vector (Sigma) and verified by sequence analysis before transfection, respectively. The MDA-MB-231 and Hs578T cells were transfected with an empty vector or pFLAG-CMV-ERβ1 by Lipofectamine 2000 (Invitrogen, USA) following selection by 1 mg/ml G418 (Gibco). AR shRNA (shAR) and scrambled shRNA control (shNC) were cloned into the pGpU6/GFP/Neo vector purchased from GenePharma (Shanghai, China) and transfected into cells according to the manufacturer’s protocol. The shRNA sequences were as follows: shAR, 5’-CACCAATGTCAACTCCAGGAT-3’, and shNC, 5’-AGTGCACGTGCATGTCCTA-3’.

### Wound-healing and Transwell assays

Cells were seeded in 6-well plates and incubated to generate confluent cultures. Wounds were scratched in the cell monolayer using a 200 μl sterile pipette tip, and cells were rinsed with PBS. The migration of the cells at the edge of the scratch was photographed at 0 and 24 h. The invasion ability of cells was determined using 24-well transwell chambers (Costar, USA) coated with matrigel (BD Biosciences, San Jose, CA). After transfection, approximately 1 × 10^5^ cells/200 μl were resuspended in medium without serum and plated in the top chamber of each transwell, and 800 μl of medium supplemented with 10% FBS was injected into the lower chamber. After 24 h incubation, the inserts were fixed with 100% methanol, subsequently stained with crystal violet and photographed under a microscope.

### Western blot and Immunofluorescence

Cells lysates were resolved by SDS-PAGE electrophoresis (30 μg/sample) and electro-transferred onto polyvinylidene fluoride (PVDF) membranes. After incubation in blocking buffer, the membranes were probed overnight at 4 °C with the following primary antibodies: GAPDH (CST), 1:5000; ERβ1(sc-6822) (Santa Cruz), 1:200; AR (D6F11) (CST), 1:2000; E-cadherin(24E10) (CST),1:1000; N-cadherin(D4R1H) (CST), 1:1000; ZEB1(D80D3) (CST), 1:1000; Snail (C15D3) (CST), 1:1000; and Twist (ab50581) (Abcam), 1:500. The subsequent steps were performed as previously described [20].

Cells were fixed with 3% paraformaldehyde for 10 min, permeabilized with 0.1% SDS solution in PBS for 10 min and then blocked for 20 min. Fixed cells were stained with primary antibodies and then with a secondary antibody coupled to Dylight 649 for 30 min. Cell nuclei were stained with Dapi-Fluoromount-G for 15 min. The expression was defined as follows: −, no immunofluorescence; ±, weak immunofluorescence; +, moderate immunofluorescence; ++, strong immunofluorescence; and +++, very strong immunofluorescence. The samples with scores ++ or +++ were defined as high expression, and the remaining samples were defined as low expression.

### Immunohistochemistry

Following deparaffinization, sections were rehydrated and subjected to antigen retrieval using citrate buffer (BioGenex, USA). The slides were incubated with primary antibodies, including ERβ1(ab27720) (Abcam) and other antibodies as described in the previous section, at 4 °C overnight. The following steps were performed as previously described [20]. The cut-offs for positivity at 1% and 20% were defined as AR-positive and ERβ1-positive, respectively [9, 21]. The percentages of positive cells and staining intensities were scored as previously described [20]. IHC scoring was performed without prior knowledge of the clinical response.

### Chromatin immunoprecipitation

The cells were treated with 1% formaldehyde for 8 min to crosslink histones to DNA. After washing with cold PBS, the cell pellets were resuspended in lysis buffer and sonicated for 8 s 7 times. The lysate was divided into three fractions, which were incubated with IgG antibody as a negative control, RNA polymerase II antibody as a positive control, or AR antibody (ab74272) (Abcam), at 4 °C overnight. To collect the immunoprecipitated complexes, protein A-Sepharose beads (Pharmacia Biotech) were added and incubated for 1 h at 4 °C. After washing, the beads were treated with RNase (50 μg/ml) for 30 min at 37 °C and then proteinase K overnight. The crosslinks were reversed by heating the sample at 65 °C for 1.5 h. DNA was extracted by the phenol/chloroform method, ethanol-precipitated, and resuspended, and it was then used for PCR, the primer sequences were shown in Table 1.

**Table 1:** Primers for ERβ1 promoter sequence in ChIP assay

ERβ1-F1: 5’-TCATAAACTTTGTGGCTAAAACAG-3’ ERβ1-R1: 5’-AGAGAAGAGGGAGGCAAG-3’
ERβ1-F2: 5’-CTCTATTTTTAAGGGTGCTTGTG-3’ ERβ1-R2: 5’-GCTATTTTCTTTTATTTTGTGGCAC-3’
ERβ1-F3: 5’-CTCAGCAAGGCAAATTTACTCTTTC-3’ ERβ1-R3: 5’-CAAGACAGCCAAGAAATCACC-3’
ERβ1-F4: 5’-TGTCTTGCCTGAGCACAGCA-3’ ERβ1-R4: 5’-CGTGCCATTACACTCCAGC-3’
ERβ1-F5: 5’- ATCTTGGCTTACTGCAACCTC-3’ ERβ1-R5: 5’-CCACCGTTAGTAATATTGTAAATGTC-3’
ERβ1-F6: 5’-GCATTGTTCATTATTGCCGGAAAC-3’ ERβ1-R6: 5’-GTATTTTTAGTAGAGACGGGGTTTC-3’
ERβ1-F7: 5’-CAAAATTAGCCAGGCGTGG-3’ ERβ1-R7: 5’-TCCTTACAAGCCCATTGCTTTC-3’
ERβ1-F8: 5’-GAACTTGGTTCTTGTTGAACATCC-3’ ERβ1-R8: 5’-ATCTCAGCCTGCCACAC-3’
ERβ1-F9: 5’-ATCTGCCTCCTTGTTCCCG-3’ ERβ1-R9: 5’-GCCCTTACTTCCTTTTCCCTTAAG-3’
ERβ1-F10: 5’-CCTTAAGGGAAAAGGAAGTAAGGGC-3’ ERβ1-R10: 5’-CCTCTCCCTGATTGGCTCGAAT-3’

### Tumor metastasis model

All animal experiments were conducted in accordance with the Guide for Care and Use of Laboratory Animal, and all experimental protocols were approved by the Animal Ethics Committee. The control and ERβ1-expressing MDA-MB-231 and Hs578T cells (1× 10^7^cells/ml, 100ul/mouse) were injected into 6-week-old BALB/c female nude mice via the tail vein to establish a tumor lung metastasis model. The lungs of mice were removed on the eighth week after injection.

### Statistical analysis

Data from at least three independent experiments are presented as the means ± standard error of the mean. Differences between groups were calculated by Student’s t‑test or one‑way analysis of variance using the SPSS 19.0 software package (SPSS Inc.). The association in TNBC tissues was explored by the Spearman rank correlation. *P* < 0.05 was considered to indicate a statistically significant difference.

## Results

### ERβ1 suppresses cell migration and invasion of AR-positive TNBC cells

**Fig. 1:**
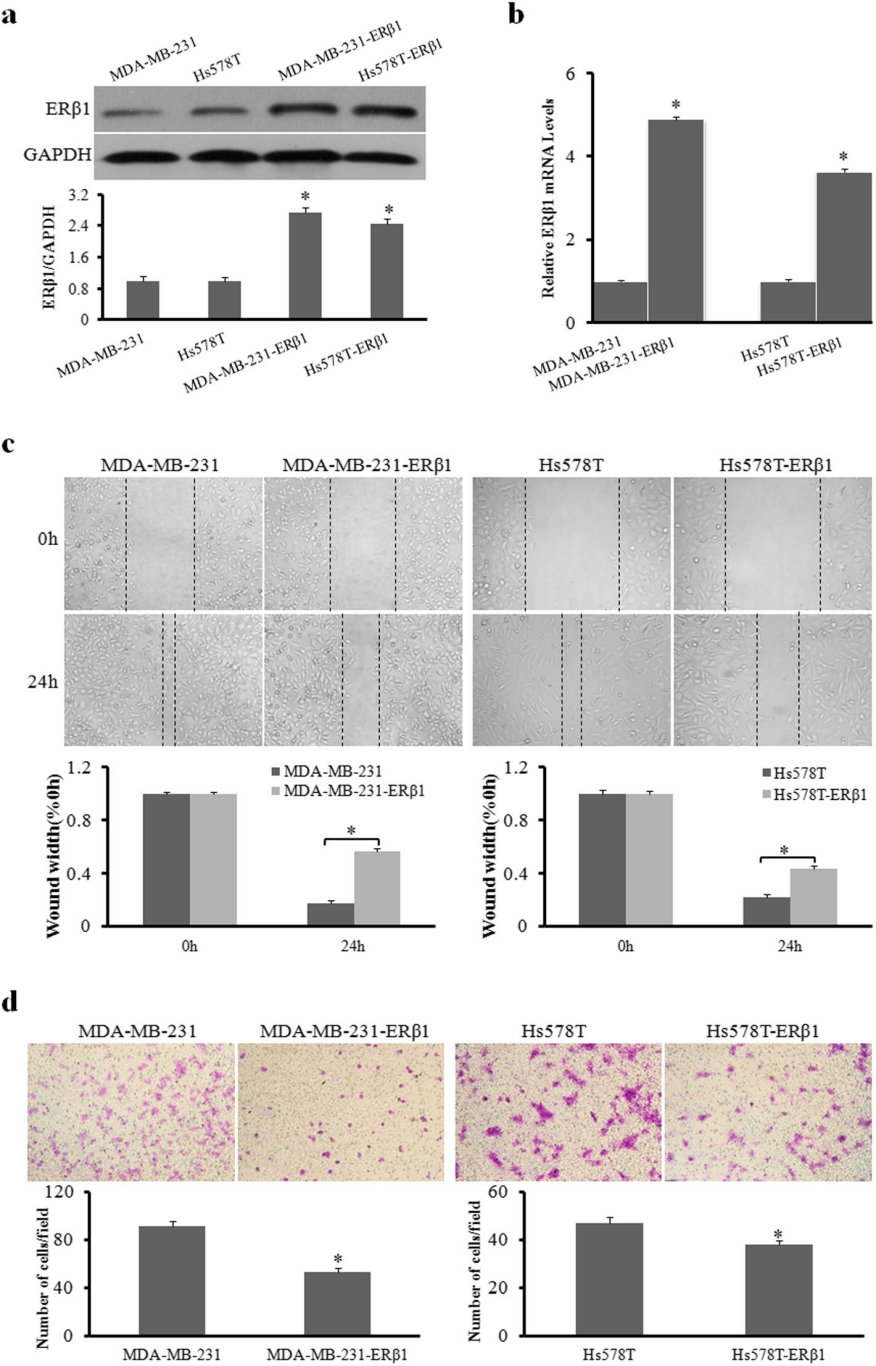
ERβ1 suppresses cell migration and invasion of AR-positive TNBC cells. **a, b** Western blot analysis and qRT-PCR of ERβ1 protein and mRNA expression in the control and stable ERβ1-transfected cell lines. **c** Representative images of wound-healing assays in the control and ERβ1-expressing cell lines. The widths of injury lines made in cells were examined at 0 and 24 h. Wound-healing migration is represented by the widths of injury lines. **d** Representative results of transwell assays showing the effect of ERβ1 overexpression on the invasion ability in control and ERβ1-expressing MDA-MB-231 and Hs578T cells. *p < 0.05

Previous studies had shown an association of ERβ1 expression with tumor metastasis and the suppression of factors known to be involved in bone metastasis in prostate cancer [22]. We hypothesized that ERβ1 may regulate metastasis in TNBC. To explore the functions of ERβ1 on tumor metastasis in AR-positive TNBC, we transfected two AR-positive TNBC cell lines, MDA-MB-231 and Hs578T, with the empty vector or the ERβ1 expression vector. Eight weeks later, stable ERβ1-expressing cell lines were obtained and confirmed by Western blot analysis and qRT-PCR (Fig. 1a and b). Wound-healing and transwell assays were then performed to evaluate the migration and invasion of these cells. The results showed that ERβ1 repressed the migration and invasion of two cell lines, MDA-MB-231-ERβ1 and Hs578T-ERβ1, when compared to the control (*P <* 0.05) (Fig. 1c and d). Our results suggested that ERβ1 inhibits the migration and invasion abilities of AR-positive TNBC cells.

### ERβ1 suppresses metastasis of AR-positive TNBC by inhibiting ZEB1

**Fig. 2:**
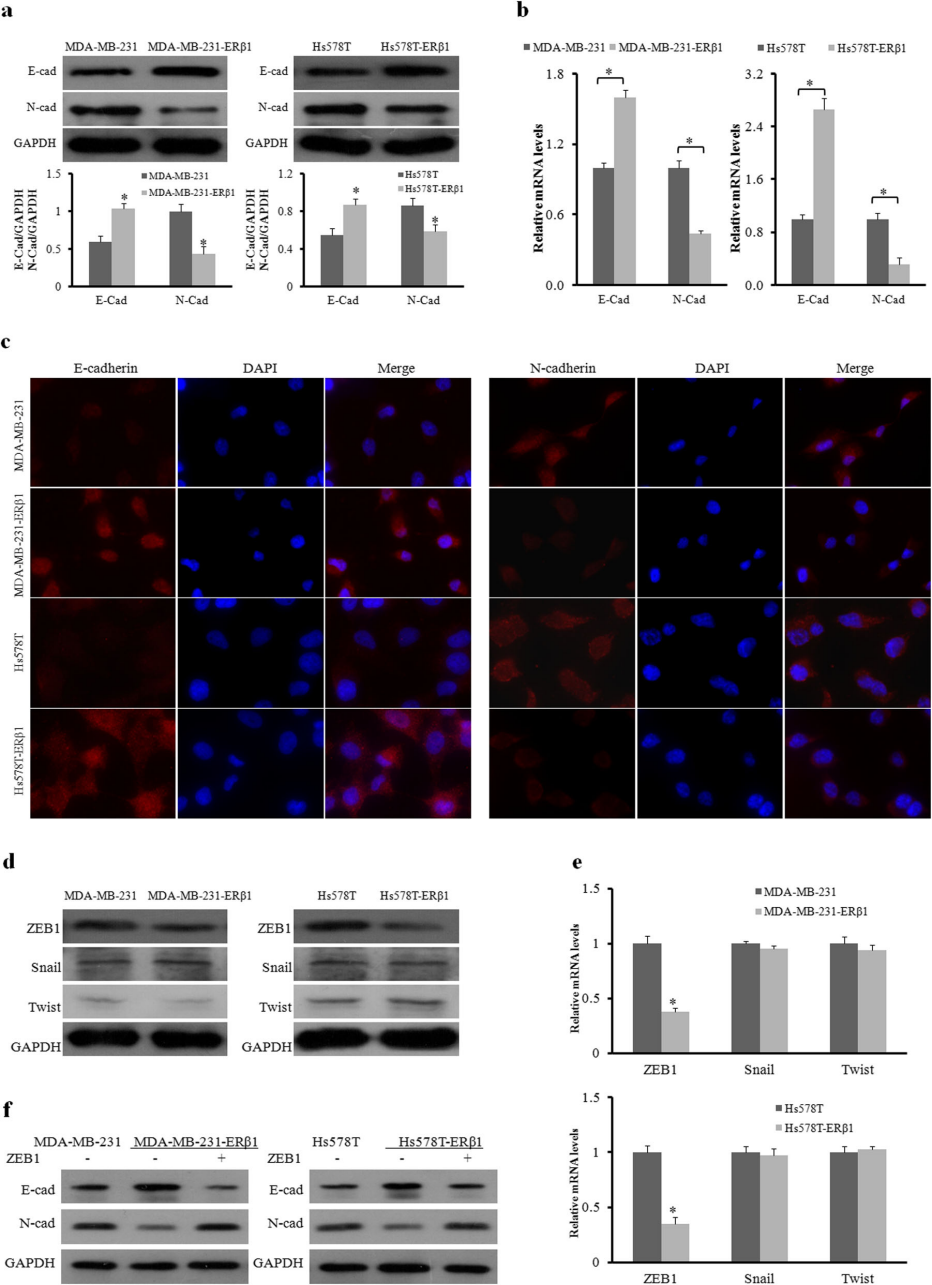
ERβ1 suppresses metastasis of AR-positive TNBC by inhibiting ZEB1. **a** Western blot analysis of E-cadherin and N-cadherin protein in control and ERβ1-expressing cells. GAPDH expression was used as the loading control. **b** qRT-PCR detection of E-cadherin and N-cadherin mRNA expression in control and ERβ1-expressing MDA-MB-231 and Hs578T cells. **c** Fluorescent microscopy analysis of the expression of E-cadherin and N-cadherin by immunofluorescence. The red signal represents E-cadherin or N-cadherin protein, and the blue signal represents the nuclear DNA staining by DAPI. **d**, **e** Western blot and qRT-PCR analyses of ZEB1, Snail and Twist in the control and ERβ1-expressing cells. **f** Western blot of E-cadherin and N-cadherin expression after transfection with ZEB1 or empty vector in the control and ERβ1-expressing MDA-MB-231 and Hs578T cells. *p < 0.05

We next investigated the underlying mechanisms by which ERβ1 suppresses the metastasis of AR-positive TNBC. As shown in Fig. 2a, compared with the control cells, ERβ1 upregulated E-cadherin epithelial marker expression (1.75-fold and 1.60-fold increase) and downregulated N-cadherin mesenchymal marker expression (56% and 32% decrease) in MDA-MB-231-ERβ1 and Hs578T-ERβ1 cells, respectively. The results of qRT-PCR revealed that ERβ1 was also positively associated with E-cadherin mRNA and inversely associated with N-cadherin mRNA (Fig. 2b). Moreover, the results of immunofluorescence showed higher expression of E-cadherin and lower expression of N-cadherin on the cell surface of ERβ1-expressing cells compared to control cells (Fig. 2c), suggesting that ERβ1 promoted cell-cell adhesion by regulating the expression of E-cadherin and N-cadherin. Several transcription factors, such as Snail, Twist and ZEB1, had been reported to promote EMT in multiple tumors, including breast cancer [23, 24]. For example, BCL6 induced EMT by promoting the ZEB1-mediated transcription repression of E-cadherin in breast cancer cells. Therefore, we examined if the inhibition of these factors was involved in the regulation of ERβ1-induced E-cadherin and N-cadherin expression. Only ZEB1 expression was reduced in both ERβ1-expressing cell lines compared to control cells (Fig. 2d). In addition, qRT-PCR revealed that ERβ1 inhibited ZEB1 expression at the transcriptional level (Fig. 2e). ZEB1 overexpression abrogated the increase of E-cadherin expression and the decrease of N-cadherin expression caused by ERβ1 overexpression (Fig. 2f). Taken together, these data showed that ERβ1 regulates E-cadherin and N-cadherin expression through inhibiting ZEB1.

### ERβ1 inhibits metastasis of AR-positive TNBC cells in vivo

**Fig. 3:**
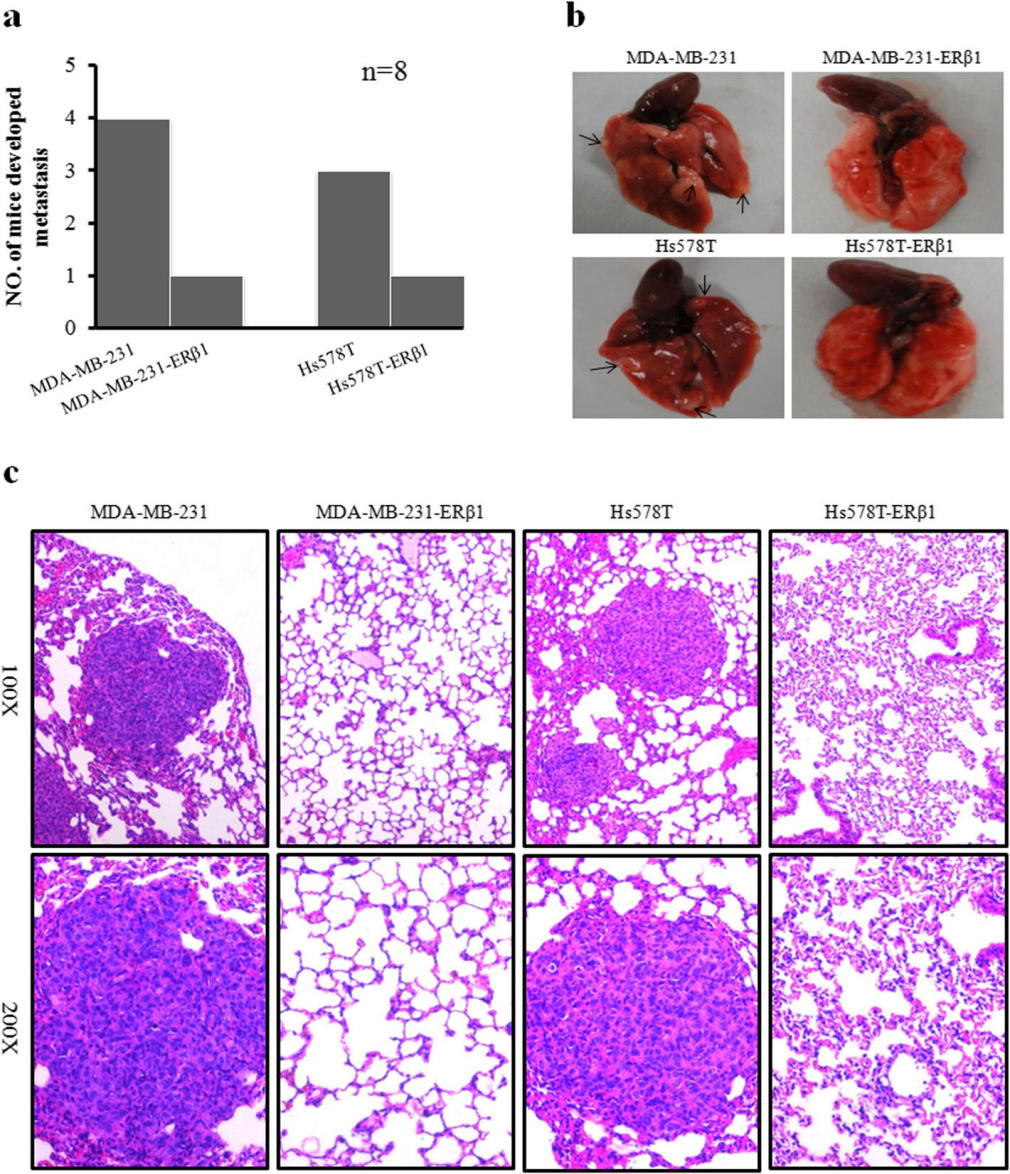
ERβ1 inhibits metastasis of AR-positive TNBC cells in vivo. **a** Lung metastasis rate of mouse models in the control and ERβ1-expressing groups (*n* = 8). **b** Representative images of lung metastasis in mouse models injected with control and ERβ1-expressing MDA-MB-231 and Hs578T cells. **c** Representative HE staining of lung sections from the control or ERβ1-expressing groups

To further investigate the anti-metastatic effect of ERβ1 in vivo, we established a lung metastasis mouse model using control TNBC cells and ERβ1-expressing TNBC cells. Lung metastasis was determined by comprehensive analysis of macroscopical observation and HE staining of lung tissue sections. The results showed that there was higher occurrence of lung metastasis (4/8 in MDA-MB-231 and 3/8 in Hs578T) in the control cells and a lower occurrence of lung metastasis (1/8 in MDA-MB-231-ERβ1 and 1/8 in Hs578T-ERβ1) in ERβ1-expressing cells (Fig. 3a and b). Fig. 3c showed the representative HE staining of lung tissues from mice treated with control or ERβ1-expressing MDA-MB-231 and Hs578T cells. Our results showed that ERβ1 inhibits metastasis of AR-positive TNBC cells in vivo.

### AR promotes the anti-metastatic effect of ERβ1

**Fig. 4:**
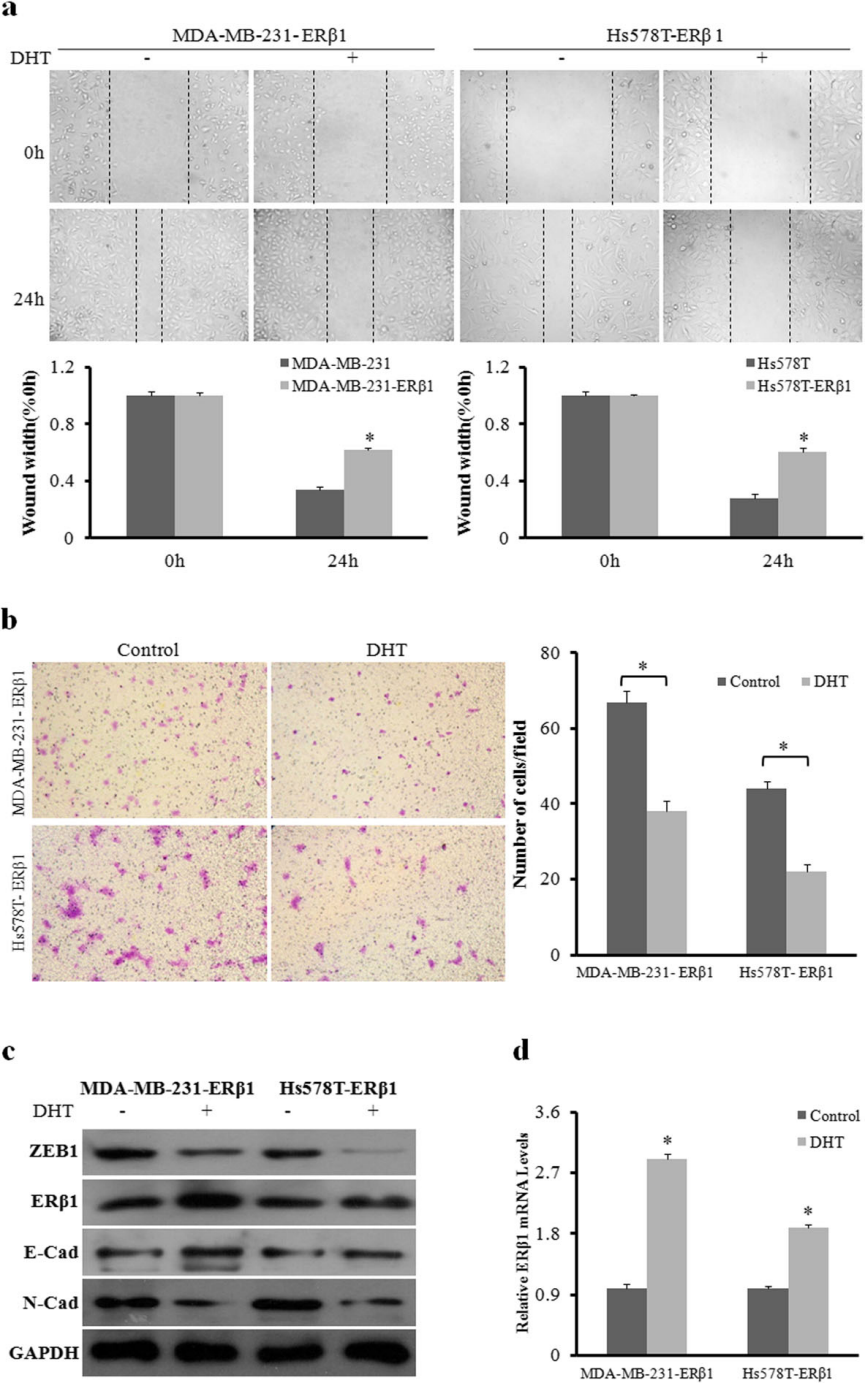
AR promotes the anti-metastatic effect of ERβ1. **a** Wound-healing assay for the cell migration of ERβ1-expressing MDA-MB-231 and Hs578T cells with or without DHT (10nM) treatment. The percentage of wound healing was calculated. **b** Representative images of transwell assays for the invasion of MDA-MB-231 and Hs578T cells treated with or without DHT. **c** Western blot analysis of ZEB1, ERβ1, E-cadherin and N-cadherin protein levels in ERβ1-expressing MDA-MB-231 and Hs578T cells after treatment with or without DHT. **d** qRT-PCR detection of ERβ1 mRNA in ERβ1-expressing MDA-MB-231 and Hs578T cells with or without DHT treatment. *p < 0.05

AR is expressed in both MDA-MB-231 and Hs578T TNBC cell lines. It has been reported that AR expression correlates with metastases in TNBC. We further examined if AR is involved in the regulation of ERβ1-mediated metastasis inhibition in TNBC cells. Wound-healing and transwell assays showed that AR activation by DHT inhibited cell migration and invasion ability in both MDA-MB-231-ERβ1 and Hs578T-ERβ1 cells compared to control cells (Fig. 4a and b). Moreover, ZEB1 protein was downregulated after AR activation, which was accompanied by upregulated E-cadherin and downregulated N-cadherin, in both ERβ1-expressing cell lines (Fig. 4c). As mentioned above, ZEB1 expression responded to the modulation of ERβ1. We measured the expression of ERβ1 after AR activation and found that activated AR increased ERβ1 protein and mRNA expression levels (Fig. 4c and d). These data suggested that AR activation enhances ERβ1 expression, which improves the suppressive effect of ERβ1-mediated metastasis in ERβ1-expressing TNBC cells.

### AR acts as a transcription factor that binds to the promoter of ERβ1

**Fig. 5:**
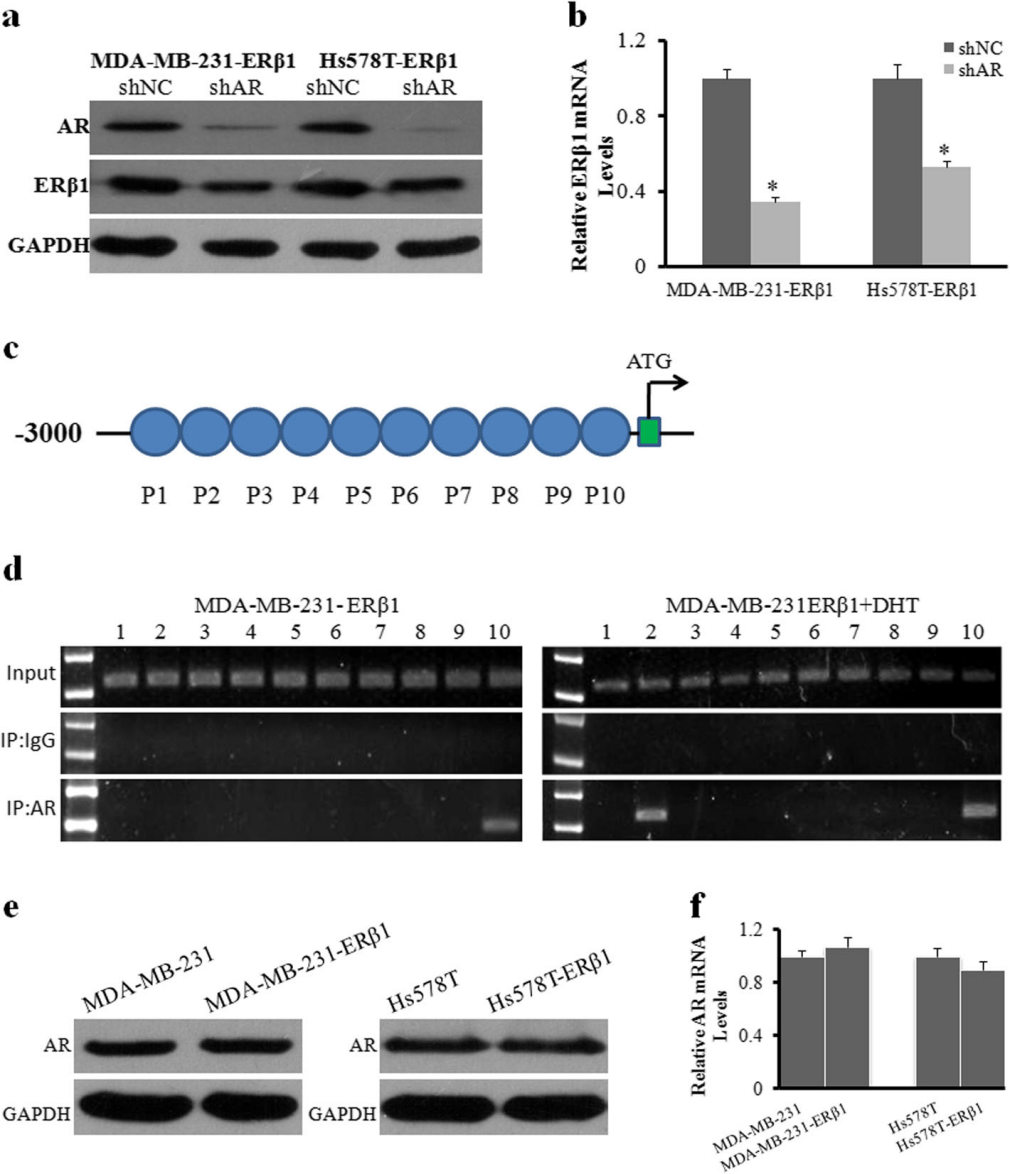
AR acts as a transcription factor that binds to the promoter of ERβ1. **a**, **b** Western blot and qRT-PCR analyses of ERβ1 protein and mRNA with or without AR knockdown in ERβ1-expressing MDA-MB-231 and Hs578T cells in the presence of DHT. **c** Primers used for PCR in the ChIP assay. **d** The gel electrophoresis of ChIP assay showed a binding site located in primer 10 without DHT treatment and two binding sites located in primers 2 and 10 with DHT treatment in ERβ1-expressing MDA-MB-231 cells. **e**, **f** Western blot and qRT-PCR analyses of AR protein and mRNA in the control and ERβ1-expressing cells. *p < 0.05

We next investigated the possible mechanisms by which AR regulated ERβ1 expression. First, we detected the expression of ERβ1 after AR knockdown by shAR in the presence of DHT. AR knockdown led to a decrease in the expression of ERβ1 protein and mRNA compared to the negative control (Fig. 5a and b), which indicated that DHT caused the above changes through activation of the AR signaling pathway. It is well known that AR, as a transcription factor, regulates the expression of many genes in many different cellular processes [25, 26]. Because we showed that ERβ1 expression was regulated by activated AR at both protein and mRNA levels, we hypothesized that AR functioned as a transcription factor that directly bound to the promoter region of ERβ1. To better determine if AR binds to the promoter of ERβ1, a ChIP assay was performed using ERβ1-expressing MDA-MB-231 cells exposed or not exposed to DHT. The results showed that AR, as a transcription factor, directly bound to the promoter of ERβ1 and that the recruitment of AR to the promoter of ERβ1 was increased with DHT treatment (Fig. 5c and d). Furthermore, no difference in AR protein and mRNA expression was observed between the control and ERβ1-expressing TNBC cells (Fig. 5e and f). Our results suggested that activated AR promotes the expression of ERβ1 by functioning as a transcription factor that directly binds to the promoter of ERβ1.

### Relationships of ERβ1 with clinicopathological factors in TNBC

**Fig. 6:**
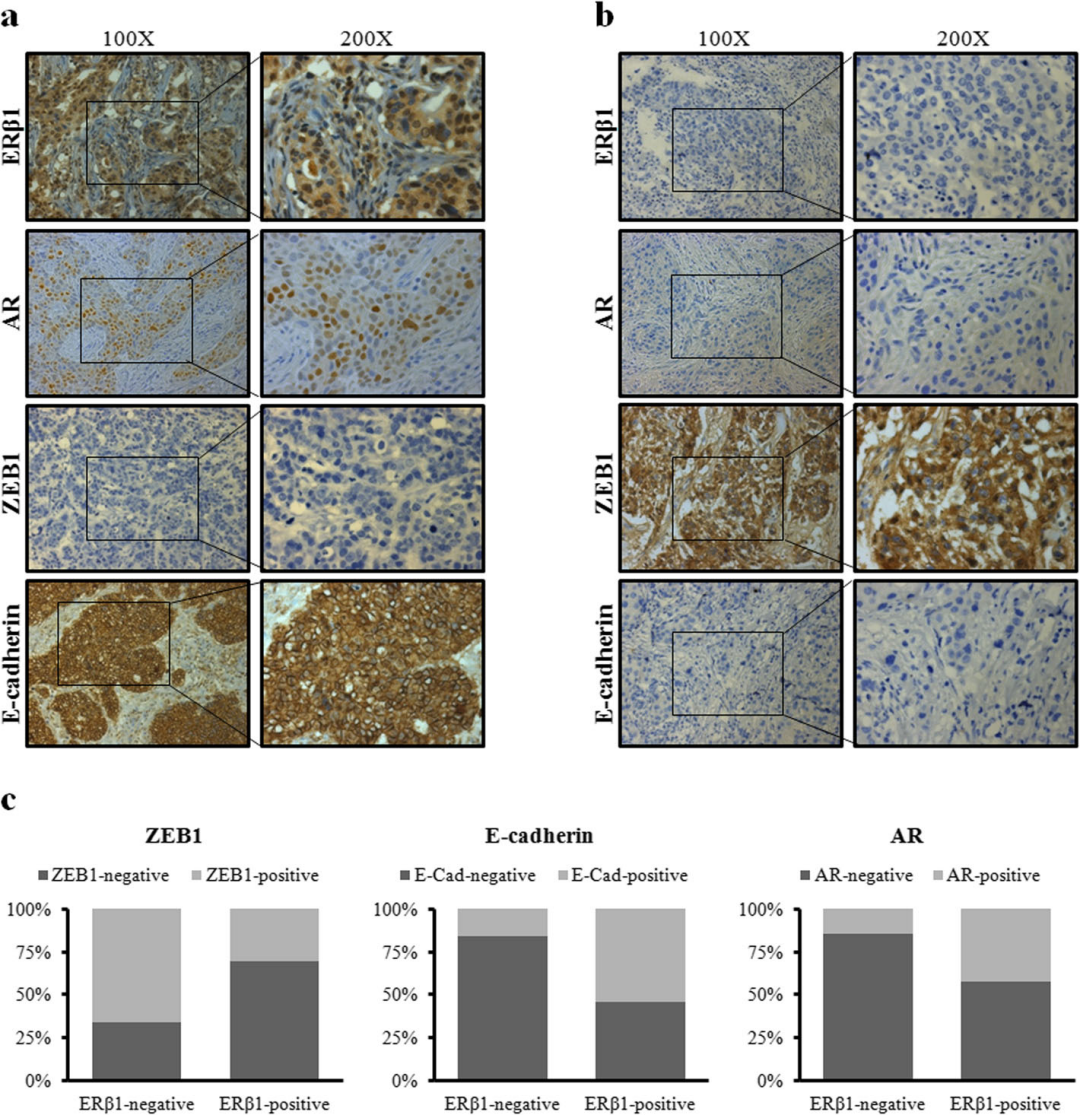
Relationships of ERβ1 with clinicopathological factors in TNBC. **a**, **b** Representative IHC staining of ERβ1 high-, AR high-, ZEB1 low-, and E-cadherin high-expression (**a**) or ERβ1 low-, AR low-, ZEB1 high-, and E-cadherin low-expression (**b**) in TNBC samples. **c** Expression ratio of AR, ZEB1 and E-cadherin in ERβ1-positive group or ERβ1-negative group, respectively

We next examined the correlation of ERβ1 with AR, ZEB1, E-cadherin and clinical characteristics in 82 TNBC samples. The protein levels of ERβ1, AR, ZEB1, and E-cadherin were determined by IHC (Fig. 6a and b). Our results showed that ERβ1 and AR were overexpressed in 31.7% (26/82) and 23.2% (19/82) of the TNBC clinical samples, respectively (Table 2). There was a negative correlation between ERβ1 and ZEB1 (r = −0.330, *P* = 0.003) and a positive correlation between ERβ1 and E-cadherin (r = 0.391, *P* < 0.001) (Table 2). Moreover, ERβ1 expression was negatively correlated with lymph node metastasis (r = −0.368, *P* = 0.001), and positively correlated with the expression of AR (r = 0.309, *P* = 0.005). There was no association of ERβ1 expression with age and tumor size. Besides, the 82 TNBC clinical samples were divided into ERβ1-negative or ERβ1-positive groups based on the level of ERβ1. The ERβ1-positive group had higher percentage of E-cadherin-positive, ZEB1-negative and AR- positive samples (Fig. 6c).

**Table 2:** Patient characteristics and relationships between ERβ1 and clinicopathologic factors

Variables	ERβ1-Negative		ERβ1-Positive		r *P*
	No. of patients (*n* =56)	%	No. of patients (*n* = 26)	%	
Age(years)					−0.036 0.749
≤48	28	50.00	14	53.85	
>48	28	50.00	12	46.15	
Tumor size(cm)					0.009 0.936
≤2	22	39.29	10	38.46	
2–5	32	57.14	15	57.69	
>5	2	3.57	1	3.85	
Metastatic lymph nodes					−0.368 0.001*
Negative	15	26.79	17	65.38	
Positive	41	73.21	9	34.62	
AR					0.309 0.005*
Negative	48	85.71	15	57.69	
Positive	8	14.29	11	42.31	
ZEB1					−0.330 0.003*
Negative	19	33.93	18	69.23	
Positive	37	66.07	8	30.77	
E-cadherin					0.391 0.0001*
Negative	47	83.93	12	46.15	
Positive	9	16.07	14	53.85	

*P < 0.05

## Discussion

Numerous findings have shown that TNBC is a heterogeneous disease not only on the clinical level but also on the molecular level [2]. TNBC is associated with a significantly higher probability of relapse and metastasis compared with other breast cancer subtypes [27]. The molecular complexity of TNBC has led to the sub-classification into different subgroups, which is necessary to better identify molecular-based therapies [28, 29]. For example, the AR signaling pathway has long been thought to play a critical role in TNBC and to likely be relevant to tumor metastasis. According to AR status, TNBC is divided into two subtypes as follows: AR-positive TNBC or Quadruple Negative breast cancer [4, 30]. Interestingly, it also has been reported that ERβ1 is involved in the regulation of metastasis in breast cancer. For example, some studies have shown that ERβ1-positive TNBC patients tend to be less likely to develop lymphatic metastasis [12]. ERβ1 represses EMT by destabilizing EGFR in basal-like breast cancer [31]. However, other studies have indicated that ERβ1 shows no correlation with metastasis and vascular invasion in breast cancer [14].

Here, we examined if ERβ1 influences migration and invasion of AR-positive TNBC cells and explored potential mechanisms. We found that ERβ1 inhibited migration and reduced the invasiveness of AR-positive TNBC cells. The expression of EMT markers, such as E-cadherin and N-cadherin, has been reported to correlate with tumor metastasis [32, 33]. For example, PTK6 inhibition suppresses metastases of TNBC via Snail-dependent E-cadherin regulation [23]. We examined if ERβ1 inhibits invasion and migration by regulating EMT markers. ERβ1 was found to regulate the expression of E-cadherin by inhibiting its transcriptional repressor, ZEB1, in AR-positive TNBC cells. When control and ERβ1-expressing TNBC cells were injected into nude mice, the ERβ1-expressing cells were less likely to form lung metastases, suggesting that ERβ1 functions as an important anti-metastasis factor.

Published studies focusing on the correlation between AR expression and tumor metastasis in TNBC remain controversial. Decreased AR expression has been reported to associate with the occurrence of distant metastasis [9]. Additionally, AR negativity has been associated with a shorter disease-free interval and overall survival (OS) compared to AR-positive TNBCs [34]. However, other studies have found that AR-positive TNBC is more common in older patients and has a higher propensity for lymph node metastases, and they have found that AR promotes cell migration in TNBC cells [7, 35]. In our study, AR activation enhanced the inhibitory effect of ERβ1 on metastasis in ERβ1-expressing TNBC cells, and the protein and mRNA expression of ERβ1 was altered when AR was activated by DHT or knocked down by shAR. AR promoted the expression of ERβ1 by functioning as a transcription factor that directly bound to the promoter of ERβ1. Moreover, the expression of AR was not altered in control or ERβ1-expressing cells. By examining clinical TNBC specimens, we found that the expression of ERβ1 was negatively correlated with ZEB1 expression and positively correlated with E-cadherin expression. The AR-positive TNBC specimens had a higher percentage of ERβ1-positive samples compared to the AR-negative TNBC specimens.

There were several defects in the present study. For instance, the sample size was small, and there was a lack of complete information of clinical features. In addition, clinical samples originating from the metastasis lesion and not the primary lesion may be more relevant for the aim of this study. Future research should be performed to identify the binding site where AR binds to the promoter of ERβ1. Furthermore, follow-up records should be completed to allow correlation analyses with progression-free survival (PFS) and OS.

## Conclusions

**Fig. 7:**
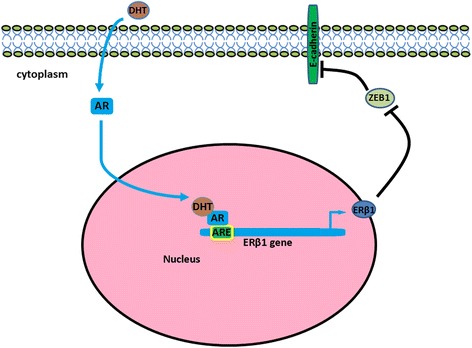
Proposed working model of activated AR regulation of the transcription of ERβ1, which subsequently suppresses ZEB1

In summary, our work indicated that ERβ1 can inhibit metastasis of AR-positive TNBC through suppressing ZEB1. ERβ1 may play an important role in repressing invasive behavior and inhibiting metastasis in this subset of TNBC. Activated AR promotes ERβ1-induced metastasis inhibition of ERβ1-expressing TNBC cells (Fig. 7). The results provided the probable mechanism behind the anti-metastatic effect of ERβ1 and strengthened the possibility that ERβ1 can help to identify patients with AR-positive TNBC with lower risk to develop metastasis.

## Abbreviations

AR: Androgen receptor
ATCC: American Type Culture Collection
DHT: Dihydrotestosterone
EMT: Epithelial-mesenchymal transition
ERα: Estrogen receptor alpha
ERβ1: Estrogen receptor beta 1
ERβ2: Estrogen receptor beta 2
FBS: Fetal bovine serum
HER2: Human epidermal growth factor receptor 2
IHC: Immunohistochemistry
PR: Progesterone receptor
shAR: AR shRNA.
shNC: shRNA control
TNBC: Triple-negative breast cancer
